# Trends in the Evaluation and Treatment of Patients Presenting With Sigmoid Volvulus Over the Past 15 Years

**DOI:** 10.7759/cureus.94438

**Published:** 2025-10-13

**Authors:** Daniel L Cohen, Rozan Hasona, Yona Rothé, Refael Aminov, Adi Eindor-Abarbanel, Haim Shirin, Anton Bermont, Vered Richter

**Affiliations:** 1 The Gonczarowski Family Institute of Gastroenterology and Liver Diseases, Shamir Medical Center, Zerifin, ISR; 2 The Gray Faculty of Medical and Health Sciences, Tel Aviv University, Tel Aviv, ISR; 3 Internal Medicine, Shamir Medical Center, Zerifin, ISR; 4 Pediatric Gastroenterology, Shamir Medical Center, Zerifin, ISR

**Keywords:** c-reactive protein, ct scan, intestinal volvulus, laparoscopy, sigmoid colon

## Abstract

Background: Few studies have assessed changes over time in the evaluation and treatment of patients presenting with acute sigmoid volvulus. We sought to evaluate trends in sigmoid volvulus care at a single center between 2010 and 2024. We hypothesized that there would be increasing rates in the use of computed tomography (CT) scans, lactate levels, laparoscopic surgery, and same-admission surgery.

Methods: A single-center, retrospective study was performed for all adult admissions for acute sigmoid volvulus over 15 years (2010-2024). Each admission was assessed individually for demographic, clinical, laboratory, radiographic, endoscopic, and surgical data. As each admission was assessed separately and was the unit of analysis, some patients could contribute multiple episodes to the study. Outcomes were compared between three five-year periods (2010-2014, 2015-2019, 2020-2024).

Results: One hundred and eighty-four episodes of sigmoid volvulus from 103 unique patients were included (median age 77.0 [IQR 66.2-83.0], 76.6% male). CT scan use increased from 17.6% to 25.7% to 47.6% (p=0.001 groups 1 vs 3; p=0.009 groups 2 vs 3; p trend <0.001). While the rate in which lactate was checked did not significantly change over time (p trend 0.634), the rate in which C-reactive protein (CRP) was assessed increased from 29.4% to 75.7% to 92.1% (p<0.001 for groups 1 vs 2; p<0.001 for groups 1 vs 3; p=0.011 for groups 2 vs 3; p trend <0.001). The rates of same-admission surgery (13.7% to 21.7% to 23.8% of all cases; p trend=0.193) and laparoscopic surgery (0% to 13.3% to 20.0% of all same-admission surgeries; p trend=0.219) trended upwards but were not statistically significant. Patient outcomes, including mortality (p trend=0.747), did not significantly change during the study period.

Conclusions: CT scans and CRP are increasingly being used, while surgical trends are progressing more slowly in the management of sigmoid volvulus patients, although these changes have not been associated with detectable changes in outcomes. Limited power and confounding may obscure small effects and have limited our findings. Larger, multi-center studies may help to better elucidate these trends.

## Introduction

Colonic volvulus occurs due to the twisting of a segment of colon on its mesentery. It is the third leading cause of colonic obstruction globally [1]. Most cases (60-75%) occur in the sigmoid colon, with risk factors including dolicho-sigmoid, diabetes, neuropsychiatric disease, institutional placement, and prolonged bed rest [1].

Several recent guidelines have addressed the appropriate evaluation and treatment of patients presenting with sigmoid volvulus, including those from the World Society of Emergency Surgeons (WSES), American Society of Colon and Rectal Surgeons (ASCRS), and American Society for Gastrointestinal Endoscopy (ASGE) [1-3]. While generally in agreement, there are some slight differences between these guidelines. For example, in terms of initial evaluation, the ASCRS guidelines recommend that a “basic laboratory assessment” be performed, while the WSES specifically states that blood gas and lactate should be checked. In terms of imaging, both the ASCRS and WSES state that a plain abdominal X-ray can be used as the initial diagnostic test, while the ASGE states that X-ray has been replaced by computed tomography (CT) scan and advocates for CT as the preferred diagnostic tool. 

All three guidelines recommend endoscopic detorsion in patients presenting with sigmoid volvulus in the absence of signs of perforation or ischemia. While the WSES and ASGE guidelines recommend placement of a colonic decompression tube after successful endoscopic detorsion and decompression, the ASCRS says this is optional. After successful detorsion, all three guidelines recommend that surgical intervention be performed as soon as possible, even during the index admission. The decision as to whether the resection should be performed laparoscopically or open is left up to the surgeons.

Few studies have investigated trends in how sigmoid volvulus is evaluated and treated over time. Longitudinal studies are important as advances in surgical techniques and access to advanced imaging modalities may impact patient outcomes. One study showed an increase in the use of laparoscopic surgery and a decrease in mortality over time [4]. Another suggests that endoscopic detorsion has become more successful [5]. No studies have assessed the usage of CT scans or lactate levels. Specifically, prior studies have not quantified CT scan or lactate utilization trends over time, which has motivated this study.

We, therefore, aimed to evaluate trends in the evaluation and treatment of sigmoid volvulus at our institution over the past 15 years with a specific focus on areas where the optimal evaluation and treatment are not clearly agreed upon, namely the use of CT scans for diagnosis, the assessment of lactate levels, and the surgical approach performed. We hypothesized that the rates of CT scan usage, checking lactate levels, and laparoscopic surgery have all increased over the study period.

## Materials and methods

Study design

This was a single-center, retrospective study performed at a large, tertiary-referral medical center. The electronic medical records were searched for all adult patients (age 18 or older) admitted with the diagnosis of volvulus from 2010 through 2024. The search was performed according to the International Classification of Diseases, Ninth Revision (ICD-9), code 560.2 (volvulus). As the study institution only uses ICD-9 (and does not use ICD-10), ICD-10 was not used. Charts were reviewed for demographic, clinical, laboratory, radiographic, endoscopic, and surgical data. 

Cases were included if sigmoid volvulus was confirmed radiographically (by either CT scan or abdominal X-ray). Cases were excluded if sigmoid volvulus was not confirmed, including cases of dolicho-sigmoid without volvulus, colonic pseudo-obstruction, fecal impaction, cecal volvulus, and gastric volvulus. If sigmoid volvulus was suspected radiographically but another diagnosis was found endoscopically, then the case was excluded. For patients with multiple episodes of sigmoid volvulus, each admission was assessed individually and included if it met the criteria. Thus, some patients may have multiple episodes included in the study. A patient’s initial admission with sigmoid volvulus was considered their index admission.

Sigmoid volvulus management protocol at the study institution

At the study institution, there is no formalized protocol for the evaluation and management of sigmoid volvulus patients. These patients are admitted to a general surgery service. Decisions as to the care of the patient are made by the attending surgeon on the case, although physicians are expected to practice according to the accepted guidelines in the field [1-3]. In areas where there is an accepted consensus amongst guidelines and experts, that is what is performed. For example, in uncomplicated cases, flexible sigmoidoscopy with endoscopic detorsion is performed emergently. In complicated cases with peritonitis, perforation, ischemia, or failed endoscopic detorsion, emergency surgical resection is performed.

For areas where there is no agreed-upon consensus in the guidelines, these decisions are made by the attending physician. Thus, decisions including whether to place a rectal tube, when to perform non-emergent surgery, and what type of surgery to perform, are left up to the treating physician based upon the patient’s clinical status and physician's expertise.

The fact that the institution does not have a formalized protocol allowed this research study to be performed. If the physicians could only follow a set protocol, then it would be impossible to assess changes in their management of sigmoid volvulus over time, since they would not have any freedom to change their management decisions based on what they believe is the most appropriate, up-to-date, and best care to provide.

Clinical data

Radiology reports were reviewed for all cases. All CT and X-ray studies were interpreted by a senior attending radiologist to confirm the diagnosis of sigmoid volvulus.

For patients who presented to the Emergency Department (ED) with sigmoid volvulus, laboratory data were taken from their initial ED labs. For those diagnosed with sigmoid volvulus while already hospitalized, laboratory data were taken from their daily labs on the day of diagnosis. Thus, laboratory results were obtained within 24 hours of sigmoid volvulus diagnosis. Evaluated laboratory data included white blood cell count (WBC), absolute neutrophil count, neutrophil percentage, lactate, and C-reactive protein (CRP).

For cases in which endoscopic detorsion was performed, the endoscopy reports were reviewed to confirm the diagnosis of sigmoid volvulus, evaluate the technical success of the endoscopic detorsion, and evaluate for signs of ischemia. All endoscopy procedures were performed by a senior attending gastroenterologist. If multiple endoscopies were performed during the same admission, only the results of the first detorsion are included. Cases performed on weekend days or between 3 pm and 7 am were considered “on-call.”

Surgical reports were reviewed for the urgency of the operation, the operative findings, and the type of procedure performed. The urgency of the surgery was defined as either semi-elective, urgent, or emergent. Laparoscopic cases converted to open surgery were counted as open. The decision on whether to place a rectal tube during admission was the sole decision of the admitting surgeon.

Outcomes and comparisons

The 15-year time period from 2010 to 2024 was divided into three sub-periods of five years each (group 1: 2010-2014; group 2: 2015-2019; group 3: 2020-2024). These five-year periods were compared to each other in terms of various outcomes. The primary outcomes assessed included: whether a lactate level was checked; the use of a CT scan for diagnosis; and the surgical approach (laparoscopic or open) performed. Additional assessed outcomes included: whether a CRP level was checked; whether a rectal tube was placed on admission; whether endoscopic detorsion was performed and if it was successful; whether definitive surgery was performed during the same admission; inpatient and 30-day all-cause mortality rates; length of hospitalization; and 30-day readmission rate. In addition, as has previously been described, a “combined serious outcomes” variable was used, which included all cases of ischemia (whether on CT or endoscopy), urgent or emergent surgery, and inpatient or 30-day mortality [6].

These outcomes were assessed for all episodes of sigmoid volvulus. Additionally, as index admissions may be evaluated and treated differently than patients with a known prior history of sigmoid volvulus, a subgroup analysis including only index cases was performed. 

Ethical considerations

The study was approved by the medical center’s Institutional Review Board (approval #0007-25-ASF on 16 January 2025). The study was exempted from the need for informed consent as the research was retrospective and all data were de-identified.

Statistical analyses

Categorical variables were reported as numbers and percentage. Since all continuous variables had a skewed distribution per histogram, they were reported as medians with interquartile range (IQR). Comparisons of categorical variables were performed using the Chi-square test and Fisher’s Exact test. Comparisons involving continuous variables were performed using the Mann-Whitney U test. For evaluating trends between the three time periods, Chi-square trend tests were performed. For all statistical calculations, a p-value <0.05 was considered statistically significant. The data was analyzed using IBM SPSS Statistics for Windows, Version 26 (Released 2019; IBM Corp., Armonk, New York, United States).

## Results

The overall study population

**Figure 1:**
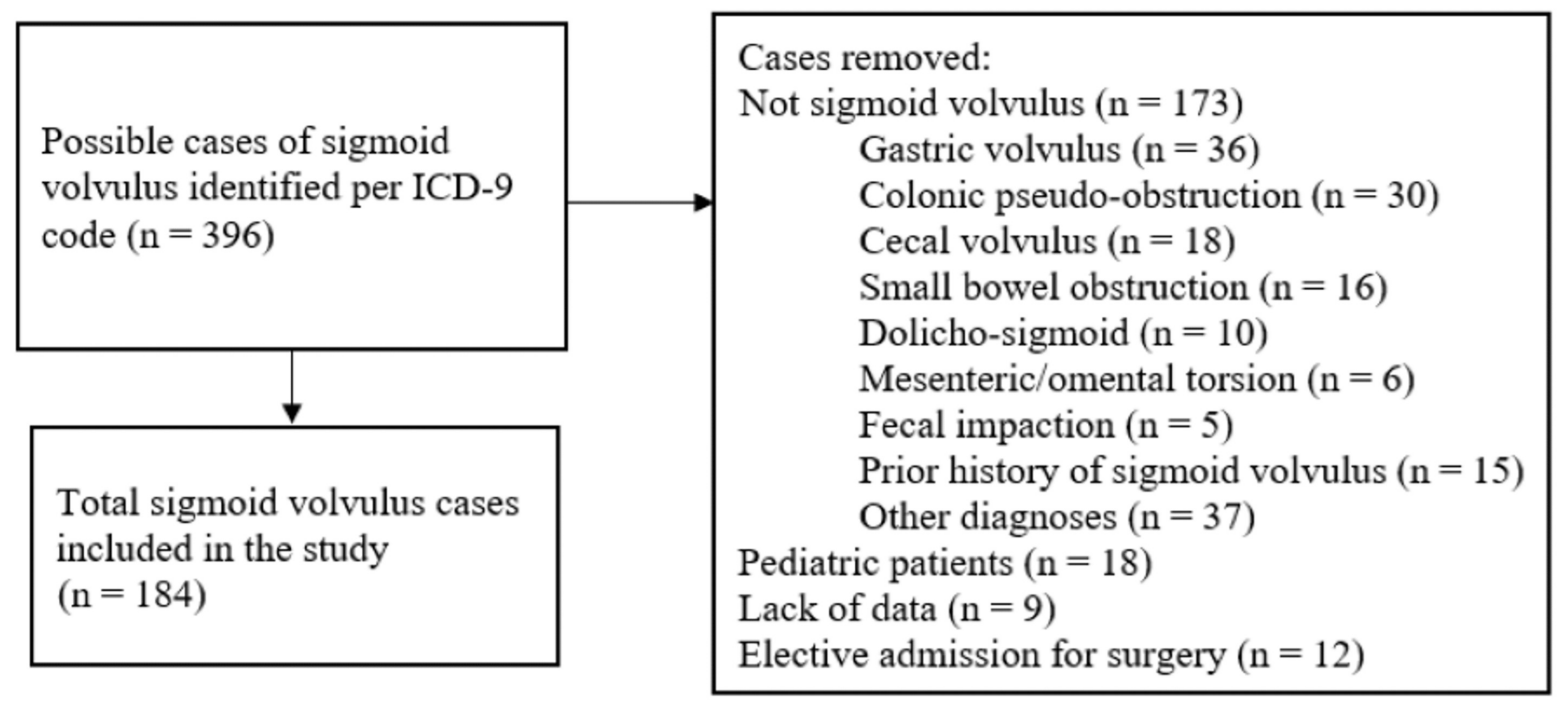
Flowchart of the cases included in the study ICD-9: International Classification of Diseases, Ninth Revision.

Per ICD-9 search, a total of 396 possible episodes of sigmoid volvulus were identified. After excluding cases not meeting the inclusion criteria, a total of 184 episodes were included in the study (Figure 1). These episodes came from 103 unique patients.

Demographic and clinical data of the study population can be found in Table 1. The median age was 77.0 (IQR 66.2-83.0) with 76.6% male. Ninety-six cases (52.2%) represented their index admission for sigmoid volvulus. Neurological disease was common (56.5%), including 39.1% with dementia and 16.8% with a prior stroke.

**Table 1:** Demographics and clinical history of the study population Comparisons of categorical variables were performed using the chi-square test and Fisher’s exact test.  Comparisons involving continuous variables were performed using the Mann-Whitney U test.  For all statistical calculations, a p-value <0.05 was considered statistically significant.

	Overall	Group 1: 2010-2014	Group 2: 2015-2019	Group 3: 2020-2024			
	n=184	n=51	n=70	n=63	p 1vs2	p 1vs3	p 2vs3
Demographics							
Age	77.0 [66.2-83.0]	78.0 [72.0-83.0]	77.0 [46.2-82.2]	74.0 [60.0-84.0]	0.119	0.182	0.506
Male gender	141 (76.6%)	41 (80.4%)	55 (78.6%)	45 (71.4%)	0.807	0.269	0.341
Index admission	96 (52.2%)	33 (64.7%)	31 (44.3%)	32 (50.8%)	0.026	0.136	0.453
Nursing home resident	24 (13.0%)	7 (13.7%)	9 (12.9%)	8 (12.7%)	0.889	0.872	0.978
Medical history							
Prior abdominal or pelvic surgery	41 (22.3%)	6 (11.8%)	19 (27.1%)	16 (25.4%)	0.039	0.067	0.819
Ischemic heart disease	50 (27.2%)	21 (41.2%)	16 (22.9%)	13 (20.6%)	0.031	0.017	0.757
Congestive heart failure	11 (6.0%)	5 (9.8%)	2 (2.9%)	4 (6.3%)	0.131	0.496	0.422
Diabetes mellitus	70 (38.0%)	18 (35.3%)	28 (40.0%)	24 (38.1%)	0.598	0.758	0.822
Neurologic disease	104 (56.5%)	33 (64.7%)	36 (51.4%)	35 (55.6%)	0.145	0.322	0.634
Dementia	72 (39.1%)	27 (52.9%)	20 (28.6%)	25 (39.7%)	0.007	0.158	0.176
Parkinson's disease	32 (17.4%)	18 (35.3%)	9 (12.9%)	5 (7.9%)	0.003	<0.001	0.356
Mental retardation	6 (3.3%)	1 (2.0%)	5 (7.2%)	0 (0%)	0.189	0.447	0.059
Epilepsy	14 (7.7%)	3 (6.0%)	8 (11.6%)	3 (4.8%)	0.354	0.999	0.156
Alzheimer's disease	13 (7.1%)	5 (9.8%)	3 (4.3%)	5 (7.9%)	0.279	0.751	0.476
Stroke	31 (16.8%)	12 (23.5%)	10 (14.3%)	9 (14.3%)	0.193	0.206	0.999
Psychiatric disease	4 (2.2%)	2 (3.9%)	0 (0%)	2 (3.2%)	0.176	0.999	0.222

Overall patient evaluation

The evaluation of sigmoid volvulus patients can be seen in Table 2. 

**Table 2:** Evaluation of sigmoid volvulus patients including imaging, laboratory data, and endoscopic detorsion CT scan: computed tomography scan; WBC: white blood cell; CRP: C-reactive protein. Comparisons of categorical variables were performed using the chi-square test and Fisher’s exact test. Comparisons involving continuous variables were performed using the Mann-Whitney U test. For all statistical calculations, a p-value <0.05 was considered statistically significant.

	Overall	Group 1: 2010-2014	Group 2: 2015-2019	Group 3: 2020-2024			
	n=184	n=51	n=70	n=63	p-value 1 vs 2	p-value 1 vs 3	p-value 2 vs 3
Imaging confirmatory test							
Abdominal X-ray	127 (69.0%)	42 (82.4%)	52 (74.3%)	33 (52.4%)	0.293	0.001	0.009
CT scan	57 (31.0%)	9 (17.6%)	18 (25.7%)	30 (47.6%)			
Ischemia on imaging	11 (6.0%)	4 (7.8%)	3 (4.3%)	4 (6.3%)	0.453	0.999	0.707
Laboratory data							
WBC level	9.2 [7.4-11.7]	9.8 [7.8-11.4]	8.9 [7.4-12.4]	9.4 [7.1-11.7]	0.657	0.573	0.903
WBC assessed	181 (98.4%)	51 (100%)	69 (98.6%)	61 (96.8%)	0.999	0.510	0.603
Absolute neutrophil count	7.1 [5.3-9.6]	7.4 [6.0-9.6]	6.5 [5.0-9.5]	7.3 [5.3-9.9]	0.272	0.672	0.605
Percentage neutrophils	78.3 [69.5-85.2]	82.4 [74.4-86.0]	76.6 [64.9-85.3]	76.9 [70.1-83.6]	0.021	0.028	0.730
Lactate level	1.5 [1.1-2.3]	1.5 [1.1-2.4]	1.4 [1.0-2.3]	1.5 [1.0-2.1]	0.259	0.353	0.922
Lactate assessed	134 (72.8%)	35 (68.6%)	53 (75.7%)	46 (73.0%)	0.387	0.607	0.722
CRP level	9.5 [2.9-35.2]	33.4 [8.8-82.0]	7.6 [2.3-45.2]	9.1 [3.2-24.2]	0.086	0.020	0.939
CRP assessed	126 (68.5%)	15 (29.4%)	53 (75.7%)	58 (92.1%)	<0.001	<0.001	0.011
Rectal tube placed	85 (46.2%)	21 (41.2%)	34 (48.6%)	30 (47.6%)	0.420	0.492	0.913
Endoscopic interventions							
Endoscopy performed	132 (72.1%)	34 (66.7%)	53 (75.7%)	45 (72.6%)	0.274	0.495	0.681
On-call exam	61 (45.5%)	12 (34.3%)	19 (35.8%)	30 (65.2%)	0.881	0.006	0.004
Signs of ischemia on endoscopy	14 (10.5%)	1 (2.9%)	9 (17.0%)	4 (8.9%)	0.047	0.379	0.239
Successful endoscopic detorsion	121 (90.3%)	32 (91.4%)	50 (94.3%)	39 (84.8%)	0.679	0.502	0.181
Repeat detorsion performed during the same admission	19 (13.8%)	5 (13.9%)	6 (11.3%)	8 (16.3%)	0.751	0.758	0.463

Imaging to confirm sigmoid volvulus was performed in all cases. In 69.0%, only an abdominal X-ray was performed, while in 31.0% a CT scan was performed. In 11 cases (6.0%), signs of ischemia were identified on imaging. In terms of laboratory data, WBC counts were checked in nearly all cases (98.4%), with lactate (72.8%) and CRP (68.5%) less commonly assessed. 

A rectal tube was placed in 85 cases (46.2%). Endoscopic detorsion was performed in 132 cases (72.1%), of which it was successful in 121 of them (90.3%). In 19 cases (13.8%), a repeat endoscopic detorsion was required during the same admission. 

Overall surgical treatment and patient outcomes

Surgical and outcome data can be seen in Table 3. 

**Table 3:** Surgical interventions and clinical outcomes of the study population Comparisons of categorical variables were performed using the chi-square test and Fisher’s exact test. Comparisons involving continuous variables were performed using the Mann-Whitney U test.  For all statistical calculations, a p-value <0.05 was considered statistically significant.

	Overall	Group 1: 2010-2014	Group 2: 2015-2019	Group 3: 2020-2024	p-value 1 vs 2	p-value 1 vs 3	p-value 2 vs 3
		n=51	n=70	n=63			
Surgical interventions							
Surgery performed during the same admission	37 (20.2%)	7 (13.7%)	15 (21.7%)	15 (23.8%)	0.262	0.175	0.777
Urgency of surgery					0.533	0.387	0.896
Emergent	18 (48.6%)	5 (71.4%)	7 (46.7%)	6 (40.0%)			
Urgent	8 (21.6%)	1 (14.3%)	3 (20.0%)	4 (26.7%)			
Semi-elective	11 (29.7%)	1 (14.3%)	5 (33.3%)	5 (33.3%)			
Urgent/emergent surgery	26 (70.3%)	6 (85.7%)	10 (66.7%)	10 (66.7%)	0.616	0.616	0.999
Type of surgery					0.505	0.233	0.129
Sigmoidectomy with ostomy and Hartman pouch	20 (55.6%)	4 (66.7%)	6 (40.0%)	10 (66.7%)			
Sigmoidectomy with primary anastomosis	12 (33.3%)	1 (16.7%)	6 (40.0%)	5 (33.3%)			
Subtotal or total colectomy	4 (11.1%)	1 (16.7%)	3 (20.0%)	0 (0%)			
Laparoscopic surgery	5 (13.5%)	0 (0%)	2 (13.3%)	3 (20.0%)	0.999	0.523	0.999
Days from endoscopy until surgery	1.0 [0.0-6.0]	0.0 [0.0-4.5]	1.0 [0.0-6.5]	3.0 [0.5-6.0]	0.566	0.089	0.650
Patient outcomes							
Length of admission							
Overall	3.0 [2.0-7.2]	4.0 [2.0-7.0]	3.0 [2.0-7.2]	3.0 [2.0-8.0]	0.724	0.869	0.808
No surgery	3.0 [2.0-4.0]	3.0 [2.0-5.7]	3.0 [2.0-4.0]	3.0 [2.0-4.0]	0.296	0.350	0.914
Same-admission surgery	13.0 [8.0-23.5]	8.0 [8.0-29.0]	15.0 [8.0-30.0]	12.0 [6.0-16.0]	0.769	0.630	0.202
Discharge to a nursing home	21 (11.4%)	7 (13.7%)	8 (11.4%)	6 (9.5%)	0.705	0.483	0.721
30-day readmission	47 (25.5%)	14 (27.5%)	18 (25.7%)	15 (23.8%)	0.831	0.657	0.800
Inpatient mortality	14 (7.6%)	4 (7.8%)	6 (8.6%)	4 (6.3%)	0.999	0.999	0.748
30-day mortality	14 (7.6%)	4 (7.8%)	6 (8.6%)	4 (6.3%)	0.999	0.999	0.748
Cumulative negative outcomes	38 (20.7%)	11 (21.6%)	15 (21.4%)	12 (19.0%)	0.985	0.739	0.733

Surgery was performed during the same admission in 37 cases (20.2%) with a median of 1.0 day (IQR 0.0-6.0) until surgery. Of these, only five surgeries (13.5%) were performed laparoscopically. Only 11 surgeries were considered semi-elective (6.0% of the whole cohort, 29.7% of the surgical group), with 8 (21.6%) being urgent and 18 (46.6%) being performed emergently. The most common type of surgery was a sigmoidectomy with Hartman procedure (55.6%), while sigmoidectomy with primary anastomosis (33.3%) and subtotal/total colectomy (11.1%) were less common. In none of the cases of a primary anastomosis was a derivative stoma placed.

Overall, the inpatient and 30-day mortality rates were 7.6% with a 30-day readmission rate of 25.5%. The median length of admission was 3.0 days (IQR 2.0-7.2) with 20.7% meeting the criteria for the combined serious outcomes.

Changes in the evaluation of sigmoid volvulus over time 

Next, we divided the cohort into three groups based on date of admission (2010-2014, 2015-2019, and 2020-2024). The demographic and clinical data can be seen in Table 1. There were no significant differences between the groupings in terms of age or gender. Cases in group 1 had higher rates of index admissions, ischemic heart disease, dementia, and Parkinson’s disease, while also having lower rates of abdominal or pelvic surgery.

Data on the evaluation of patients can be seen in Table 2. In terms of imaging, there were differences over time. CT scan use increased from 17.6% to 25.7% to 47.6% (absolute increase of +30.0%). This was statistically significant comparing groups 1 vs 3 (p=0.001) and groups 2 vs 3 (p=0.009), as well as the overall trend (p<0.001). While no differences were noted in the rates at which WBC count or lactate were checked over time, the rate at which CRP was assessed increased significantly from 29.4% to 75.7% to 92.1%, an absolute increase of +62.7%. This increase was significant between each time period (p<0.001 for groups 1 vs 2; p<0.001 for groups 1 vs 3; p=0.011 for groups 2 vs 3), as well as overall (p trend <0.001). 

There were no significant changes in the rates at which rectal tubes were placed, endoscopic detorsion was performed, or the success rate of endoscopic detorsion. There was an increase in the rate of endoscopic exams being performed “on-call” in group 3 (p=0.006 vs group 1; p=0.004 vs group 2).

Changes in the surgical technique and patient outcomes over time

No significant changes were noted in terms of surgery over time (Table 3). While the rates of same-admission surgery (13.7% (7 of 51 admissions) to 21.7% (15 of 70 admissions) to 23.8% (15 of 63 admissions); p trend=0.193) and laparoscopic surgery (0% (0 of 7 surgeries) to 13.3% (2 of 15 surgeries) to 20.0% (3 of 15 surgeries); p trend=0.219) trended upwards, these differences were not statistically significant. Neither were there statistical differences in terms of the urgency of surgery or the type of surgery performed.

Additionally, no significant changes in terms of patient outcomes were noted between the time periods. The rates of mortality, 30-day readmission, and cumulative serious outcome all remained similar, as did the length of admission. The length of admission was longer for patients who underwent same-admission surgery than those who did not in each time period and overall (all p<0.001).

Subgroup analysis of index cases

Finally, we performed a subgroup analysis of index cases (96, 52.2%) of sigmoid volvulus, as these may be evaluated differently than patients with a known history of sigmoid volvulus. First, we assessed the rate of index cases in each time period and found that the percentage of index cases did not significantly change over the study period (64.7% to 44.3% to 50.8%, p=0.082).

Next, we compared the index cases to non-index admissions (88, 47.8%) for sigmoid volvulus (Table 4). 

**Table 4:** Comparison of index cases versus subsequent admissions for sigmoid volvulus CT scan: computed tomography scan; WBC: white blood cell; CRP: C-reactive protein. Comparisons of categorical variables were performed using the chi-square test and Fisher’s exact test. Comparisons involving continuous variables were performed using the Mann-Whitney U test.  For all statistical calculations, a p-value <0.05 was considered statistically significant.

	Index case	Non-index episode	
	n=96	n=88	p-value
Demographics			
Age	76.5 [68.0-83.0]	77.0 [55.5-82.7]	0.463
Male gender	68 (70.8%)	73 (83.0%)	0.052
Nursing home resident	17 (17.7%)	7 (8.0%)	0.050
Imaging confirmatory test			
Abdominal X-ray	54 (56.3%)	73 (83.0%)	<0.001
CT scan	42 (43.8%)	15 (17.0%)	
Ischemia on imaging	11 (11.5%)	0 (0%)	0.001
Laboratory data			
WBC level	9.8 [7.5-12.5]	9.0 [7.3-10.9]	0.158
WBC assessed	95 (99.0%)	86 (97.7%)	0.607
Absolute neutrophil count	7.4 [5.5-10.0]	7.0 [5.3-9.5]	0.587
Percentage neutrophils	79.4 [69.2-85.6]	76.7 [69.5-84.8]	0.262
Lactate level	1.5 [1.1-2.4]	1.5 [1.1-2.2]	0.360
Lactate assessed	68 (70.8%)	66 (75.0%)	0.526
CRP level	12.5 [2.9-36.5]	8.0 [2.9-34.9]	0.450
CRP assessed	68 (70.8%)	58 (65.9%)	0.473
Rectal tube placed	47 (49.0%)	38 (43.2%)	0.432
Endoscopic interventions			
Endoscopy performed	69 (71.9%)	63 (72.4%)	0.935
On-call exam	35 (50.0%)	26 (40.6%)	0.276
Signs of ischemia on endoscopy	10 (14.3%)	4 (6.3%)	0.136
Successful endoscopic detorsion	60 (85.7%)	61 (69.3%)	0.061
Repeat detorsion performed during the same admission	9 (12.5%)	10 (11.4%)	0.652
Surgical interventions			
Surgery performed during the same admission	24 (25.3%)	13 (14.8%)	0.077
Urgency of surgery			0.001
Emergent	17 (70.8%)	1 (7.7%)	
Urgent	3 (12.5%)	5 (38.5%)	
Semi-elective	4 (16.7%)	7 (53.8%)	
Urgent/emergent surgery	20 (83.3%)	6 (46.2%)	0.018
Type of surgery			0.165
Sigmoidectomy with ostomy and Hartman pouch	14 (58.3%)	6 (50.0%)	
Sigmoidectomy with primary anastomosis	6 (25.0%)	6 (50.0%)	
Subtotal or total colectomy	4 (16.7%)	0 (0%)	
Laparoscopic surgery	2 (8.3%)	3 (23.1%)	0.321
Days from endoscopy until surgery	0.0 [0.0-2.0]	6.0 [4.7-8.0]	<0.001
Patient outcomes			
Length of admission	4.0 [2.0-7.0]	3.0 [2.0-8.0]	0.231
Discharge to a nursing home	15 (15.6%)	6 (6.8%)	0.061
30-day readmission	21 (21.9%)	26 (29.5%)	0.233
Inpatient mortality	9 (9.4%)	5 (5.7%)	0.345
30-day mortality	9 (9.4%)	5 (5.7%)	0.345
Cumulative negative outcomes	26 (27.1%)	12 (13.6%)	0.024

There were no significant differences in terms of demographics, laboratory data assessed, rectal tube placement, or endoscopic detorsion rates. However, there was a difference in terms of imaging assessment. Index cases were significantly more likely to undergo a CT scan (43.8% vs 17.0%, p<0.001) than patients with a known history of sigmoid volvulus. Rates of ischemia on imaging were also significantly higher for index cases (11.5% vs 0%, p=0.001). Likely due to this higher rate of ischemia, index cases also had higher rates of emergent or urgent surgery (83.3% vs 46.2%, p=0.018) and a shorter time until surgery (median 0.0 vs 6.0 days, p<0.001). Cumulative serious outcomes, which includes both ischemia and emergent/urgent surgery, were also increased in index cases (27.1% vs 13.6%, p=0.024). Other surgical variables and patient outcomes, including mortality, did not differ between the groups. 

## Discussion

This study sought to evaluate trends in the evaluation and treatment of acute sigmoid volvulus over the past 15 years, focusing on previously unreported variables. We found that while the rate at which lactate was checked remained relatively stable, the use of CRP and CT scans has been significantly increasing. In addition, there have been slight, non-significant trends towards more frequent same-admission surgery and use of the laparoscopic technique.

Studies have evaluated the utility of laboratory blood tests in sigmoid volvulus. A recent study attempted to create a model to predict the presence of gangrene based on laboratory values [7]. Their model, which included WBC, CRP, lactate dehydrogenase, and potassium, had an area-under-the-curve value of 0.836. However, of these tests, only an elevated CRP value was significantly associated with gangrene on multivariate analysis [7]. The WSES guidelines specifically recommend testing for lactate levels, writing that this is “crucial,” but no studies proving the utility of this are cited. In a prior study of ours, we found a trend towards higher lactate values in patients found to have ischemia during endoscopic detorsion (3.1 +/- 2.5 vs 1.7 +/- 1.0), but this did not reach statistical significance (p=0.055) [6]. In this current study, we found a slight increase in the rate at which lactate levels were assessed, increasing from 68.6% to 75.7% and 73.0% in the subsequent periods, but this trend was not significant. 

Interestingly, while the rate at which lactate was checked did not significantly increase, the rate at which CRP was checked had a significant increase from 29.4% to 75.7% to 92.1%. CRP, like lactate, is not specific for sigmoid volvulus or the presence of ischemia, but it has been shown to correlate with poor outcomes in patients hospitalized in general hospital wards [8-11]. As CRP can be helpful in many conditions, it is not surprising that its use is increasing in many different patient populations [12], just as we found here with sigmoid volvulus patients. The studies showing the utility and benefits of CRP in other conditions tend to be more recently published [8-11]. Additionally, older studies of sigmoid volvulus (published during the beginning years of our study period) did not mention or recommend CRP testing [13-15]. These factors likely explain why CRP is being assessed more frequently for sigmoid volvulus now than 15 years ago. As a prior study showed that CRP is associated with the presence of gangrene in sigmoid volvulus [7], this may be an even more important laboratory value to check than lactate levels, although we did not find any correlation to better patient outcomes with the increased use of CRP over time. 

CT scans can accurately diagnose different forms of colonic volvulus [16]. Additionally, they are now freely being used to diagnose sigmoid volvulus and may be able to predict recurrence [17]. As CT scans are more frequently performed for all indications [18,19], it is not surprising that we found their use significantly increased in the diagnosis of sigmoid volvulus, increasing from 17.6% to 25.7% to 47.6%. Interestingly, we also found that CT was more frequently used during index cases, when the patient had no prior history of sigmoid volvulus. However, when these patients return, it appears that only an abdominal x-ray is needed to confirm recurrence. While the utilization of CT increased, this did not translate into detectable improvements in patient outcomes. Thus, prospective evaluation of the diagnostic yield of CT with respect to outcomes should be performed to justify the additional radiation, cost, and resource use.

Surgery is often recommended during the index admission, given the high likelihood of recurrence along with good patient outcomes when performed during the same admission [1,2,20]. However, this is not clearly recommended in the guidelines and is left up to the treating surgeon. In our study, we found a slight trend towards more patients undergoing semi-elective sigmoidectomy during the same admission, with rates increasing from 2.0% to 7.1% to 7.9% of the overall population presenting with sigmoid volvulus. However, this trend was not significant, and semi-elective surgery during the same admission remained an outcome only achieved in a small fraction of our cases.

Related to the issue of same-admission surgery is the discussion of surgical technique. As laparoscopy is a newer surgical technique, it is not surprising that its use in sigmoid volvulus was rare decades ago. Atamanalp found that all 80 sigmoid resections were performed open prior to 2002, while 10 of 21 (47.6%) were performed laparoscopically between 2002 and 2011 [21]. Guidelines leave the decision on whether to perform surgery open or laparoscopic up to the surgeon based upon surgical and patient factors [1,2], and this is based on studies showing similar outcomes between the techniques [22,23]. In our study, the rates of laparoscopic surgery were low, although they did increase from 0% to 13.3% to 20.0% during the study period. While this trend was not statistically significant as in prior studies [4,21], it does suggest an increasing use of the laparoscopic technique. 

In terms of outcomes, we did not identify any detectable differences in outcomes. Notably, there were no improvements in mortality rates (7.8% to 8.6% to 6.3%), length of admission (4.0 [2.0-7.0] to 3.0 [2.0-7.2] to 3.0 [2.0-8.0]), or combined serious outcomes (21.6% to 21.4% to 19.0%). This may be due to low mortality rates to begin with and limited power.

While this study benefited from its large number of patients, it does have some limitations. It was a single-center study, so some of the results may not be generalizable to other locations, especially as surgical expertise and management may differ between hospitals. It relied on retrospectively collected data. As discharged patients may have continued their care at other medical centers, we cannot comment on their follow-up care, including details of elective surgeries that may have been performed after discharge. Also, given its retrospective nature and patients following up at other institutions, detailed data on surgical complications and outcomes is not possible. While a large number of cases were included, the absolute number of cases for certain outcomes, such as patients undergoing laparoscopic or emergency surgery, was low and, therefore, this limited our ability to identify slight changes in practice. Finally, we must note the non-independence of including repeated episodes per patient and the indication bias inherent in lab and imaging orders.

## Conclusions

In conclusion, we identified several novel changes in which patients with acute sigmoid volvulus are being evaluated and treated. CT scans and CRP are increasingly being used to diagnose and evaluate patients. While there are trends towards more laparoscopic and same-admission surgeries, these rates remain low. The increased CT and CRP use coexisted with stable short-term outcomes in this cohort. The detection of small effects may have been limited by sample size and other limitations. Future larger studies evaluating the optimal care of sigmoid volvulus patients in which better patient outcomes are obtained are needed. 
